# 5-Bromo-3-(4-fluoro­phenyl­sulfin­yl)-2-methyl-1-benzofuran

**DOI:** 10.1107/S1600536810016181

**Published:** 2010-05-08

**Authors:** Hong Dae Choi, Pil Ja Seo, Byeng Wha Son, Uk Lee

**Affiliations:** a Department of Chemistry, Dongeui University, San 24 Kaya-dong Busanjin-gu, Busan 614-714, Republic of Korea; b Department of Chemistry, Pukyong National University, 599-1 Daeyeon 3-dong, Nam-gu, Busan 608-737, Republic of Korea

## Abstract

In the title compound, C_15_H_10_BrFO_2_S, the O atom and the 4-fluoro­phenyl group of the 4-fluoro­phenyl­sulfinyl substituent are located on opposite sides of the plane through the benzofuran fragment; the 4-fluoro­phenyl ring is approximately perpendicular to this plane [dihedral angle = 89.38 (6)°]. In the crystal, mol­ecules are linked by a Br⋯Br contact [3.4816 (5) Å], and weak inter­molecular C—S⋯π [3.499 (2) Å] and C—F⋯π [3.535 (2) Å] inter­actions.

## Related literature

For the crystal structures of similar derivatives, see: Choi *et al.* (2010*a* ▶,*b* ▶). For the biological activity of benzofuran compounds, see: Aslam *et al.* (2006 ▶); Galal *et al.* (2009 ▶); Khan *et al.* (2005 ▶). For natural products with benzofuran rings, see: Akgul & Anil (2003 ▶); Soekamto *et al.* (2003 ▶).
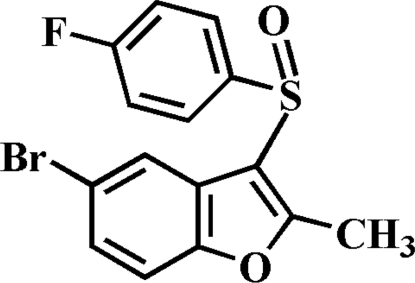


## Experimental

### 

#### Crystal data

C_15_H_10_BrFO_2_S
*M* *_r_* = 353.20
Monoclinic, 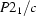

*a* = 11.4704 (4) Å
*b* = 6.1776 (2) Å
*c* = 19.6420 (7) Å
β = 98.432 (2)°
*V* = 1376.78 (8) Å^3^
*Z* = 4
Mo *K*α radiation
μ = 3.15 mm^−1^
*T* = 173 K
0.32 × 0.26 × 0.21 mm

#### Data collection

Bruker SMART APEXII CCD diffractometer
Absorption correction: multi-scan (*SADABS*; Bruker, 2009 ▶) *T*_min_ = 0.453, *T*_max_ = 0.746
11827 measured reflections
3148 independent reflections
2655 reflections with *I* > 2σ(*I*)
*R*_int_ = 0.028

#### Refinement

*R*[*F* ^2^ > 2σ(*F* ^2^)] = 0.028
*wR*(*F* ^2^) = 0.072
*S* = 1.05
3148 reflections
182 parameters
H-atom parameters constrained
Δρ_max_ = 0.36 e Å^−3^
Δρ_min_ = −0.53 e Å^−3^

### 

Data collection: *APEX2* (Bruker, 2009 ▶); cell refinement: *SAINT* (Bruker, 2009 ▶); data reduction: *SAINT*; program(s) used to solve structure: *SHELXS97* (Sheldrick, 2008 ▶); program(s) used to refine structure: *SHELXL97* (Sheldrick, 2008 ▶); molecular graphics: *ORTEP-3* (Farrugia, 1997 ▶) and *DIAMOND* (Brandenburg, 1998 ▶); software used to prepare material for publication: *SHELXL97*.

## Supplementary Material

Crystal structure: contains datablocks global, I. DOI: 10.1107/S1600536810016181/ng2767sup1.cif

Structure factors: contains datablocks I. DOI: 10.1107/S1600536810016181/ng2767Isup2.hkl

Additional supplementary materials:  crystallographic information; 3D view; checkCIF report

## Figures and Tables

**Fig. 1.**
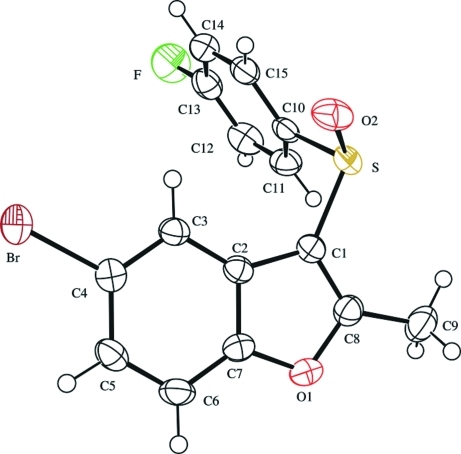
The molecular structure of the title compound with the atom numbering scheme. Displacement ellipsoids are drawn at the 50% probability level. H atoms are presented as a small spheres of arbitrary radius.

**Fig. 2.**
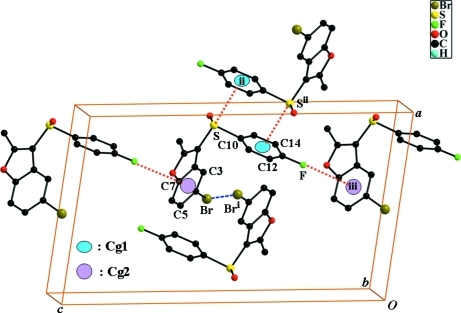
Br···Br, C–S···π and C–F···π interactions (dotted lines) in the crystal structure of the title compound. Cg denotes the ring centroid. [Symmetry codes: (i) - x + 1 , - y + 2, - z + 1; (ii) - x + 2, - y, - z + 1; (iii) x, - y + 1/2 , z - 1/2.]

## supplementary crystallographic information

### Comment

The compounds containing benzofuran skeleton show potent biological activities such as antifungal (Aslam *et al.*., 2006), antitumor and antiviral (Galal *et al.*., 2009), antimicrobial (Khan *et al.*., 2005) properties. These compounds occur widely in nature (Akgul & Anil, 2003; Soekamto *et al.*. 2003). As a part of our ongoing studies of the effect of side chain substituents on the solid state structures of 2-methyl-3-(4-fluorophenylsulfinyl)-1-benzofuran analogues (Choi *et al.*., 2010*a,b*), we report the crystal structure of the title compound (Fig. 1).

The benzofuran unit is essentially planar, with a mean deviation of 0.007 (1) Å from the least-squares plane defined by the nine constituent atoms. The 4-fluorophenyl ring is almost perpendicular to the plane of the benzofuran fragment [89.38 (6)°] and is tilted slightly towards it. The crystal packing (Fig. 2) is stabilized by a Br···Br interaction at 3.4816 (5) Å. The molecular packing (Fig. 2) is further stabilized by a weak intermolecular C–S···π interaction between the sulfur and the 4-fluorophenyl ring of an adjacent molecule, with a C1–S···Cg1^ii^ [3.499 (2) Å] (Cg1 is the centroid of the C10-C15 4-fluorophenyl ring), and by a weak intermolecular C–F···π interaction between the fluorine and the benzene ring of a neighbouring benzofuran system, with C13–F···Cg2^iii^ [3.535 (2) Å] (Cg2 is the centroid of the C2-C7 benzene ring).

### Experimental

77% 3-Chloroperoxybenzoic acid (224 mg, 1.0 mmol) was added in small portions to a stirred solution of 5-bromo-3-(4-fluorophenylsulfanyl)-2-methyl-1-benzofuran (303 mg, 0.9 mmol) in dichloromethane (30 mL) at 273 K. After being stirred at room temperature for 4h, the mixture was washed with saturated sodium bicarbonate solution and the organic layer was separated, dried over magnesium sulfate, filtered and concentrated at reduced pressure. The residue was purified by column chromatography (silica gel, hexane-ethyl acetate, 1:1 v/v) to afford the title compound as a colorless solid [yield 78%, m.p. 401-402 K; R_f_ = 0.64 (hexane-ethyl acetate, 1:1 v/v)]. Single crystals suitable for X-ray diffraction were prepared by slow evaporation of a solution of the title compound in benzene at room temperature.

### Refinement

All H atoms were positioned geometrically and refined using a riding model, with C–H = 0.93 Å for aryl and 0.96 Å for methyl H atoms. *U*_iso_(H) = 1.2*U*_eq_(C) for aryl and 1.5*U*_eq_(C) for methyl H atoms.

### Crystal data

**Table:**

C_15_H_10_BrFO_2_S	*F*(000) = 704
*M**_r_* = 353.20	*D*_x_ = 1.704 Mg m^−^^3^
Monoclinic, *P*2_1_/*c*	Mo *K*α radiation, λ = 0.71073 Å
Hall symbol: -P 2ybc	Cell parameters from 4955 reflections
*a* = 11.4704 (4) Å	θ = 2.6–27.5°
*b* = 6.1776 (2) Å	µ = 3.15 mm^−^^1^
*c* = 19.6420 (7) Å	*T* = 173 K
β = 98.432 (2)°	Block, colourless
*V* = 1376.78 (8) Å^3^	0.32 × 0.26 × 0.21 mm
*Z* = 4	

### Data collection

**Table:**

Bruker SMART APEXII CCD diffractometer	3148 independent reflections
Radiation source: rotating anode	2655 reflections with *I* > 2σ(*I*)
graphite multilayer	*R*_int_ = 0.028
Detector resolution: 10.0 pixels mm^-1^	θ_max_ = 27.5°, θ_min_ = 1.8°
φ and ω scans	*h* = −14→14
Absorption correction: multi-scan (*SADABS*; Bruker, 2009)	*k* = −8→7
*T*_min_ = 0.453, *T*_max_ = 0.746	*l* = −25→25
11827 measured reflections	

### Refinement

**Table:**

Refinement on *F*^2^	Primary atom site location: structure-invariant direct methods
Least-squares matrix: full	Secondary atom site location: difference Fourier map
*R*[*F*^2^ > 2σ(*F*^2^)] = 0.028	Hydrogen site location: difference Fourier map
*wR*(*F*^2^) = 0.072	H-atom parameters constrained
*S* = 1.05	*w* = 1/[σ^2^(*F*_o_^2^) + (0.0338*P*)^2^ + 0.5303*P*] where *P* = (*F*_o_^2^ + 2*F*_c_^2^)/3
3148 reflections	(Δ/σ)_max_ < 0.001
182 parameters	Δρ_max_ = 0.36 e Å^−^^3^
0 restraints	Δρ_min_ = −0.53 e Å^−^^3^

### Special details

**Table:**

Geometry. All esds (except the esd in the dihedral angle between two l.s. planes) are estimated using the full covariance matrix. The cell esds are taken into account individually in the estimation of esds in distances, angles and torsion angles; correlations between esds in cell parameters are only used when they are defined by crystal symmetry. An approximate (isotropic) treatment of cell esds is used for estimating esds involving l.s. planes.
Refinement. Refinement of F^2^ against ALL reflections. The weighted R-factor wR and goodness of fit S are based on F^2^, conventional R-factors R are based on F, with F set to zero for negative F^2^. The threshold expression of F^2^ > 2sigma(F^2^) is used only for calculating R-factors(gt) etc. and is not relevant to the choice of reflections for refinement. R-factors based on F^2^ are statistically about twice as large as those based on F, and R- factors based on ALL data will be even larger.

### Fractional atomic coordinates and isotropic or equivalent isotropic displacement parameters (Å^2^)

**Table:**

	*x*	*y*	*z*	*U*_iso_*/*U*_eq_	
Br	0.50266 (2)	0.78650 (4)	0.558051 (12)	0.04487 (9)	
S	0.93498 (4)	0.16534 (9)	0.60352 (3)	0.03310 (13)	
F	0.72642 (13)	0.0128 (3)	0.31650 (7)	0.0625 (4)	
O1	0.69236 (13)	0.0086 (2)	0.71739 (7)	0.0367 (3)	
O2	0.97008 (13)	0.3979 (3)	0.60830 (8)	0.0431 (4)	
C1	0.80963 (16)	0.1358 (3)	0.64457 (9)	0.0291 (4)	
C2	0.70597 (16)	0.2719 (3)	0.63776 (9)	0.0273 (4)	
C3	0.66711 (16)	0.4522 (3)	0.59916 (10)	0.0296 (4)	
H3	0.7113	0.5128	0.5680	0.035*	
C4	0.55947 (17)	0.5382 (3)	0.60907 (9)	0.0308 (4)	
C5	0.49100 (17)	0.4492 (4)	0.65478 (10)	0.0343 (5)	
H5	0.4188	0.5114	0.6594	0.041*	
C6	0.52929 (19)	0.2691 (4)	0.69335 (10)	0.0367 (5)	
H6	0.4845	0.2076	0.7241	0.044*	
C7	0.63708 (18)	0.1851 (3)	0.68398 (9)	0.0299 (4)	
C8	0.79788 (18)	−0.0166 (3)	0.69259 (10)	0.0329 (4)	
C9	0.8737 (2)	−0.1974 (4)	0.72202 (12)	0.0460 (6)	
H9A	0.9422	−0.2058	0.6993	0.069*	
H9B	0.8305	−0.3307	0.7155	0.069*	
H9C	0.8975	−0.1730	0.7703	0.069*	
C10	0.86555 (15)	0.1228 (3)	0.51655 (10)	0.0284 (4)	
C11	0.81711 (18)	−0.0770 (4)	0.49734 (11)	0.0370 (5)	
H11	0.8172	−0.1863	0.5299	0.044*	
C12	0.7688 (2)	−0.1142 (4)	0.42988 (12)	0.0436 (5)	
H12	0.7342	−0.2466	0.4164	0.052*	
C13	0.77312 (18)	0.0497 (4)	0.38319 (11)	0.0400 (5)	
C14	0.82316 (19)	0.2465 (4)	0.40008 (11)	0.0391 (5)	
H14	0.8257	0.3529	0.3668	0.047*	
C15	0.87026 (17)	0.2836 (3)	0.46836 (11)	0.0332 (4)	
H15	0.9048	0.4162	0.4815	0.040*	

### Atomic displacement parameters (Å^2^)

**Table:**

	*U*^11^	*U*^22^	*U*^33^	*U*^12^	*U*^13^	*U*^23^
Br	0.04284 (15)	0.03939 (15)	0.05249 (15)	0.01201 (10)	0.00742 (10)	0.00670 (10)
S	0.0217 (2)	0.0377 (3)	0.0401 (3)	−0.0009 (2)	0.00528 (19)	0.0033 (2)
F	0.0624 (9)	0.0825 (12)	0.0413 (7)	−0.0034 (8)	0.0035 (6)	−0.0134 (7)
O1	0.0403 (8)	0.0406 (9)	0.0311 (7)	0.0008 (7)	0.0112 (6)	0.0085 (6)
O2	0.0390 (8)	0.0431 (9)	0.0478 (9)	−0.0181 (7)	0.0088 (7)	−0.0041 (7)
C1	0.0260 (9)	0.0304 (10)	0.0309 (9)	−0.0018 (8)	0.0041 (7)	0.0009 (8)
C2	0.0252 (9)	0.0294 (10)	0.0275 (9)	−0.0034 (8)	0.0045 (7)	−0.0024 (8)
C3	0.0276 (9)	0.0298 (11)	0.0323 (9)	−0.0024 (8)	0.0076 (7)	0.0019 (8)
C4	0.0312 (10)	0.0300 (11)	0.0304 (9)	0.0005 (8)	0.0023 (8)	−0.0037 (8)
C5	0.0270 (10)	0.0450 (13)	0.0319 (10)	0.0022 (9)	0.0076 (8)	−0.0072 (9)
C6	0.0355 (11)	0.0477 (14)	0.0296 (10)	−0.0023 (10)	0.0136 (8)	0.0015 (9)
C7	0.0339 (10)	0.0317 (11)	0.0248 (9)	−0.0024 (9)	0.0063 (7)	0.0001 (8)
C8	0.0336 (11)	0.0345 (11)	0.0299 (9)	0.0004 (9)	0.0027 (8)	0.0014 (8)
C9	0.0523 (14)	0.0442 (14)	0.0402 (12)	0.0076 (11)	0.0027 (10)	0.0124 (10)
C10	0.0198 (8)	0.0293 (10)	0.0384 (10)	0.0022 (8)	0.0116 (7)	0.0008 (8)
C11	0.0366 (11)	0.0294 (11)	0.0472 (12)	−0.0027 (9)	0.0137 (9)	0.0025 (9)
C12	0.0407 (12)	0.0369 (13)	0.0549 (13)	−0.0071 (10)	0.0131 (10)	−0.0131 (11)
C13	0.0329 (11)	0.0516 (15)	0.0368 (11)	0.0042 (10)	0.0095 (9)	−0.0071 (10)
C14	0.0342 (11)	0.0430 (13)	0.0419 (11)	0.0045 (10)	0.0118 (9)	0.0077 (10)
C15	0.0259 (9)	0.0294 (11)	0.0459 (11)	−0.0003 (8)	0.0100 (8)	0.0020 (9)

### Geometric parameters (Å, °)

**Table:**

Br—C4	1.895 (2)	C6—C7	1.378 (3)
Br—Br^i^	3.4816 (5)	C6—H6	0.9300
S—O2	1.4910 (17)	C8—C9	1.480 (3)
S—C1	1.7581 (19)	C9—H9A	0.9600
S—C10	1.795 (2)	C9—H9B	0.9600
F—C13	1.360 (2)	C9—H9C	0.9600
O1—C8	1.378 (2)	C10—C15	1.379 (3)
O1—C7	1.378 (2)	C10—C11	1.383 (3)
C1—C8	1.353 (3)	C11—C12	1.379 (3)
C1—C2	1.446 (3)	C11—H11	0.9300
C2—C3	1.384 (3)	C12—C13	1.372 (3)
C2—C7	1.395 (3)	C12—H12	0.9300
C3—C4	1.384 (3)	C13—C14	1.365 (3)
C3—H3	0.9300	C14—C15	1.389 (3)
C4—C5	1.390 (3)	C14—H14	0.9300
C5—C6	1.381 (3)	C15—H15	0.9300
C5—H5	0.9300		
			
C4—Br—Br^i^	158.65 (6)	C1—C8—C9	133.19 (19)
O2—S—C1	107.60 (10)	O1—C8—C9	116.30 (18)
O2—S—C10	106.21 (9)	C8—C9—H9A	109.5
C1—S—C10	98.20 (8)	C8—C9—H9B	109.5
C8—O1—C7	106.71 (15)	H9A—C9—H9B	109.5
C8—C1—C2	107.72 (17)	C8—C9—H9C	109.5
C8—C1—S	124.30 (16)	H9A—C9—H9C	109.5
C2—C1—S	127.87 (15)	H9B—C9—H9C	109.5
C3—C2—C7	119.89 (18)	C15—C10—C11	120.69 (19)
C3—C2—C1	135.47 (17)	C15—C10—S	119.42 (16)
C7—C2—C1	104.63 (17)	C11—C10—S	119.65 (16)
C2—C3—C4	117.02 (17)	C12—C11—C10	120.0 (2)
C2—C3—H3	121.5	C12—C11—H11	120.0
C4—C3—H3	121.5	C10—C11—H11	120.0
C3—C4—C5	122.64 (19)	C13—C12—C11	118.1 (2)
C3—C4—Br	118.76 (14)	C13—C12—H12	121.0
C5—C4—Br	118.60 (15)	C11—C12—H12	121.0
C6—C5—C4	120.57 (19)	F—C13—C14	118.4 (2)
C6—C5—H5	119.7	F—C13—C12	118.2 (2)
C4—C5—H5	119.7	C14—C13—C12	123.4 (2)
C7—C6—C5	116.72 (18)	C13—C14—C15	118.1 (2)
C7—C6—H6	121.6	C13—C14—H14	120.9
C5—C6—H6	121.6	C15—C14—H14	120.9
C6—C7—O1	126.41 (17)	C10—C15—C14	119.7 (2)
C6—C7—C2	123.16 (19)	C10—C15—H15	120.2
O1—C7—C2	110.43 (17)	C14—C15—H15	120.2
C1—C8—O1	110.50 (17)		
			
O2—S—C1—C8	−130.32 (18)	C1—C2—C7—O1	0.0 (2)
C10—S—C1—C8	119.71 (19)	C2—C1—C8—O1	0.7 (2)
O2—S—C1—C2	45.3 (2)	S—C1—C8—O1	177.08 (14)
C10—S—C1—C2	−64.66 (19)	C2—C1—C8—C9	−179.4 (2)
C8—C1—C2—C3	178.8 (2)	S—C1—C8—C9	−3.1 (4)
S—C1—C2—C3	2.6 (3)	C7—O1—C8—C1	−0.7 (2)
C8—C1—C2—C7	−0.4 (2)	C7—O1—C8—C9	179.43 (18)
S—C1—C2—C7	−176.65 (15)	O2—S—C10—C15	9.31 (17)
C7—C2—C3—C4	0.1 (3)	C1—S—C10—C15	120.42 (16)
C1—C2—C3—C4	−179.1 (2)	O2—S—C10—C11	−176.30 (15)
C2—C3—C4—C5	−0.8 (3)	C1—S—C10—C11	−65.20 (17)
C2—C3—C4—Br	179.50 (14)	C15—C10—C11—C12	−2.5 (3)
C3—C4—C5—C6	0.7 (3)	S—C10—C11—C12	−176.83 (16)
Br—C4—C5—C6	−179.57 (16)	C10—C11—C12—C13	1.5 (3)
C4—C5—C6—C7	0.0 (3)	C11—C12—C13—F	179.44 (18)
C5—C6—C7—O1	179.32 (19)	C11—C12—C13—C14	0.3 (3)
C5—C6—C7—C2	−0.7 (3)	F—C13—C14—C15	179.71 (18)
C8—O1—C7—C6	−179.6 (2)	C12—C13—C14—C15	−1.1 (3)
C8—O1—C7—C2	0.4 (2)	C11—C10—C15—C14	1.7 (3)
C3—C2—C7—C6	0.6 (3)	S—C10—C15—C14	175.98 (15)
C1—C2—C7—C6	180.00 (19)	C13—C14—C15—C10	0.1 (3)
C3—C2—C7—O1	−179.40 (17)		

Symmetry codes: (i) −*x*+1, −*y*+2, −*z*+1.
